# Genome-wide association study of high-altitude pulmonary edema in a Han Chinese population

**DOI:** 10.18632/oncotarget.16362

**Published:** 2017-03-18

**Authors:** Xun Li, Tianbo Jin, Mingxia Zhang, Hua Yang, Xuewen Huang, Xiaobo Zhou, Wenchao Huang, Lipeng Qin, Longli Kang, Ming Fan, Suzhi Li

**Affiliations:** ^1^ Center of Altitude Disease, General Hospital of Tibet Military Area Command, Lhasa 850003, China; ^2^ Key Laboratory of High Altitude Environment and Genes Related to Diseases of Tibet Autonomous Region, School of Medicine, Xizang Minzu University, Xianyang, Shaanxi 712082, China; ^3^ School of Life Sciences, Northwest University, Xi’an, Shaanxi 710069, China; ^4^ Department of Brain Protection and Plasticity, Institute of Basic Medical Sciences, Beijing 100850, China

**Keywords:** high-altitude pulmonary edema (HAPE), single nucleotide polymorphisms (SNPs), genome wide association analysis (GWAS), susceptibility gene

## Abstract

A two-stage genome-wide association study (GWAS) was performed to identify and analyze genes and single nucleotide polymorphisms (SNPs) associated with high-altitude pulmonary edema (HAPE) in a Han Chinese patient population. In the first stage, DNA samples from 68 patients with recurrent HAPE were scanned using Affymetrix SNP Array 6.0 Chips, and allele frequencies were compared to those of 84 HapMap CHB samples to identify candidate SNPs. In the second stage, the 77 identified candidate SNPs were examined in an independent cohort of samples from 199 HAPE patients and 304 controls. Associations between SNPs and HAPE risk were tested using various genetic models. Of the 77 original SNPs, 7 were found to be associated with HAPE susceptibility in the second stage of the study. GO and pathway enrichment analysis of the 7 SNPs revealed 5 adjacent genes involved in various processes, including regulation of nucleoside diphosphate metabolism, thyroid hormone catabolism, and low-density lipoprotein receptor activity. These results suggest the identified SNPs and genes may contribute to the physiopathology of HAPE.

## INTRODUCTION

High altitude pulmonary edema (HAPE) is a non-cardiogenic form of pulmonary edema that develops in unacclimatized healthy individuals at altitudes above 2500–3000 m [1]. It is a potentially fatal medical condition and the most common cause of death among high-altitude illnesses [2]. However, the pathogenesis of HAPE remains poorly understood. Previous studies suggest that uneven hypoxic pulmonary vasoconstriction, pulmonary capillary damage, and increased pulmonary artery pressure play important roles in the pathogenesis of HAPE [3, 4].

HAPE is caused by the interaction of both genetic and environmental risk factors. Previous studies have shown that family history and race influence individual susceptibilities to HAPE [5]. Some people are susceptible to high-altitude pulmonary edema, whereas others are resistant to this condition [6, 7]. The prevalence of HAPE in the Han Chinese population in Tibet, which is about 0.4%~2% [8] and differs depending on age, gender, and occupation, is higher than that observed in native Tibetans. Rate of ascent, altitude reached, pre-acclimatization, and individual susceptibility are the major factors that contribute to high-altitude maladies [9]. In addition, patients who have previously developed HAPE are more likely to experience recurrence, which suggests the presence of a constitutional, and possibly a genetic, component in its etiology [10].

Several recent studies have examined the genetic basis of HAPE, focusing mainly on genetic polymorphisms in the beta2-adrenergic receptor [11], vascular endothelial growth factor [12], the renin angiotensin system [13], and pulmonary surfactant proteins A1 and A2 [14] in subjects susceptible to HAPE. Polymorphisms within these genes may explain individual variation in hypoxic responses and perhaps indicate susceptibility to high-altitude disease. However, the precise role of these genes in HAPE pathogenesis remains unclear.

To identify genetic variants across the whole genome that are specifically related to HAPE risk, we conducted a two-stage GWAS analysis in 68 patients with recurrent HAPE and in 84 HapMap CHB populations as references. We further evaluated potential associations with HAPE risk in a replication cohort with a total of 199 HAPE patients and 304 healthy controls from a Han Chinese population. While previous GWAS studies were based on case-control samples only, here we examined a large number of cases to identify genes that might be related to HAPE susceptibility.

## RESULTS

A total of 571 subjects, including 267 HAPE patients (246 males, 21 females; mean age 32.6 ± 10.7) and 304 controls (290 males, 14 females; mean age 36.2 ± 4.5), were examined in this analysis. Age distribution differed between the patient and control groups (*p* < 0.05). Participant characteristics are listed in Table 1.

**Table 1:** Basic characteristics of cases and controls in this study

Variables	Case N (%)	Control N (%)	*p*-value
Age (years)	32.6 ± 10.7	36.2 ± 4.5	< 0.005^a^
Sex			> 0.005^b^
Male	246 (92.0%)	290 (95.4%	
Female	21 (8.0%)	14 (4.6%)	
Total	267	304	

^a^*P* values were calculated from two-sided chi-square tests.

^b^*P* values were calculated by Student *t* tests.

**Figure 1:**
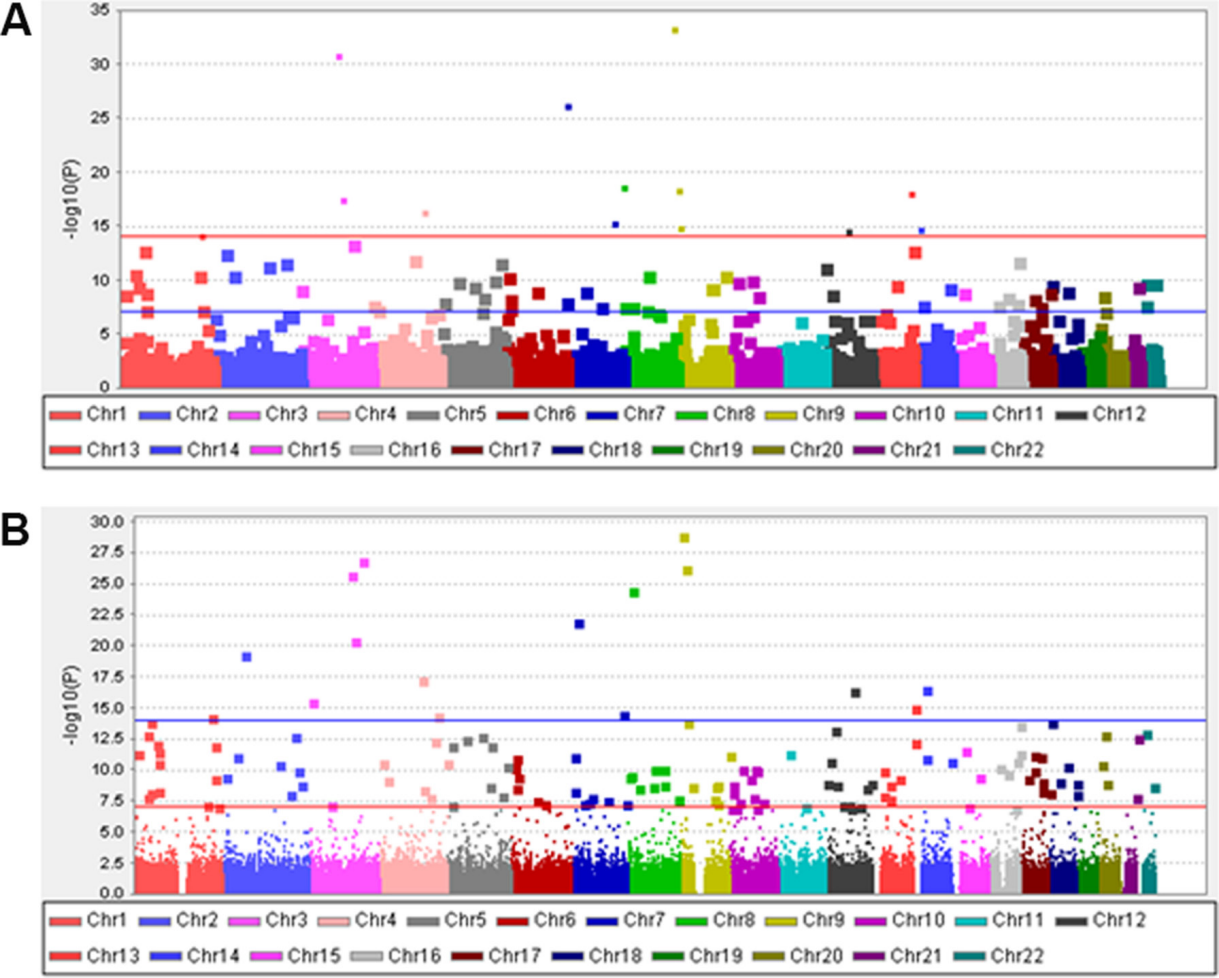
Manhattan plot for the whole SNPs in recurrent HAPE subjects of Chinese Han decent Chromosomes are shown in alternate colors. (**A**) Allelic model; (**B**) Genotypic model.

**Figure 2:**
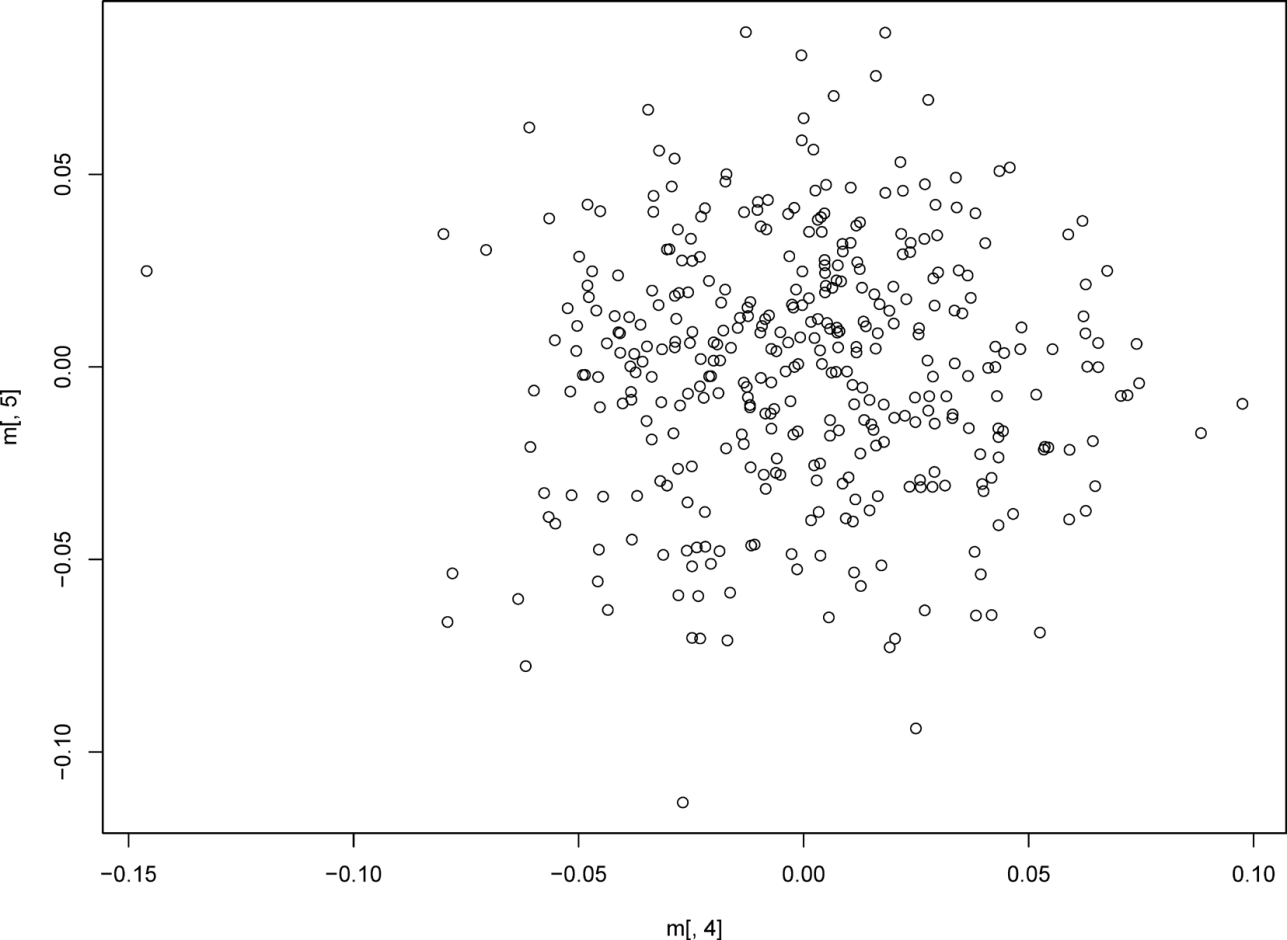
Multidimensional scaling approach (MDS) analysis for the first stage

**Figure 3:**
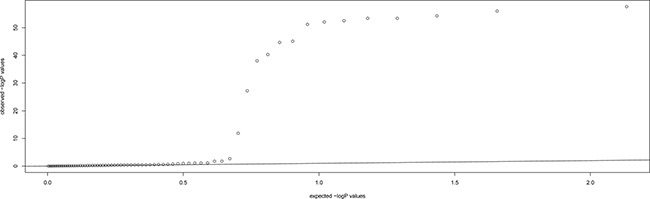
QQ plot for the whole SNPs for the first stage

We first scanned DNA samples from 68 patients with recurrent HAPE using Affymetrix Genome-Wide Human SNP Array 6.0 Chips. After filtering with standard quality-control procedures, 502,689 SNPs with an overall call rate of 99.92% were qualified for further GWAS analysis. To identify SNPs that might be associated with the risk of HAPE, we compared SNP allele frequencies in the 68 patients to those of the 84 HapMap CHB controls and found that frequencies differed for 77 SNPs. Information regarding these 77 SNPs and their associated genes is shown in Table 2. A Manhattan plot was generated for the SNPs in patients with recurrent HAPE under the allelic and genotypic model (Figure 1). MDS and QQ-plot revealed that there was no obvious population stratification in this experiment (Figure 2 and Figure 3).

**Table 2:** Basic information of the significantly different SNPs between 68 recurrent HAPE cases and 84 Hapmap CHB subjects in the first stage

SNP ID	Chromosome	Gene (s)	Alleles	MAF		Position	Band	Role
			A^a^/B	Case	Control			
rs4908427	1	CAMTA1	G/A	0.059	0.054	6976226	1p36.31	Intron
rs9661274	1		G/A	0.059	0.060	30149249	1p35.3	
rs17484974	1		C/T	0.132	0.054	39186794	1p34.3	
rs12406517	1	PPAP2B	G/C	0.110	0.072	56974278	1p32.2	Intron
rs1694212	1		T/C	0.132	0.113	61480000	1p31.3	
rs10789097	1		C/G	0.066	0.071	62119978	1p31.3	
rs17188846	1	KCNH1	C/G	0.184	0.067	211261821	1q32.2	Intron
rs2577156	1	EPRS	C/A	0.051	0.077	220190845	1q41	Intron
rs3008613	1	MIA3	G/A	0.110	0.079	222795769	1q41	Intron
rs4491711	2	RASGRP3	G/A	0.103	0.066	33776743	2p22.3	Intron
rs11125567	2	CCDC88A	A/G	0.081	0.143	55627913	2p16.1	Intron
rs11898268	2		C/A	0.000	0.071	154622125	2q23.3	
rs10167840	2		T/G	0.140	0.077	199241493	2q33.1	
rs7612512	3		G/C	0.140	0.157	3412838	3p26.2	
rs1846594	3		C/T	0.213	0.196	112916203	3q13.2	
rs11924340	3		A/G	0.060	0.110	145325196	3q24	
rs12504325	4	C4orf6	A/G	0.103	0.083	5537184	4p16.2	Downstream
rs17598758	4		G/T	0.110	0.089	20190068	4p15.31	
rs7677143	4		C/T	0.147	0.220	117082198	4q26	
rs6535838	4		A/C	0.199	0.101	153023402	4q31.3	
rs7688505	4		T/A	0.110	0.110	185828318	4q35.1	
rs41417552	5	CMBL	G/A	0.110	0.083	10305452	5p15.2	Intron
rs2161592	5		A/G	0.162	0.085	50772554	5q11.2	
rs3777207	5	ELL2	A/G	0.118	0.084	95231115	5q15	Intron
rs6595114	5		C/T	0.118	0.101	117676709	5q23.1	
rs2193963	5		C/T	0.096	0.089	121596196	5q23.2	
rs17652561	5	SLC6A7	A/G	0.162	0.185	149584197	5q32	Intron (boundary)
rs2937582	5		A/G	0.434	0.080	166465008	5q34	
rs2984100	6		C/G	0.125	0.143	8592499	6p24.3	
rs7762263	6		T/C	0.110	0.066	11975250	6p24.1	
rs4715938	6		G/C	0.103	0.113	14944857	6p23	
rs725050	6		C/T	0.162	0.196	89267376	6q15	
rs1419722	7	EIF3B	C/T	0.142	0.107	2413258	7p22.3	Intron
rs10178082	7		T/A	0.199	0.157	10706912	7p21.3	
rs4947936	7		C/A	0.103	0.133	50906752	7p12.1	
rs12226072	7		A/T	0.294	0.339	96443614	7q21.3	
rs2956956	8	DLGAP2	C/T	0.066	0.083	1553118	8p23.3	Intron
rs2980508	8	SGK223	C/T	0.096	0.106	8171732	8p23.1	Downstream
rs310282	8		C/A	0.132	0.125	23614369	8p21.2	
rs4573320	8		C/T	0.343	0.446	65128758	8q12.3	
rs1568828	8	PREX2	A/G	0.081	0.101	69122128	8q13.2	Intron
rs1006698	9	KCNV2	T/G	0.206	0.232	2725283	9p24.2	Intron
rs1011531	9		A/G	0.110	0.114	13755192	9p23	
rs13289064	9		C/G	0.228	0.179	16897685	9p22.2	
rs10984811	9	ANP32B	C/A	0.149	0.173	100784050	9q22.33	Downstream
rs12554842	9	COL5A1	T/C	0.081	0.071	137573407	9q34.3	Intron
rs11593009	10		T/A	0.051	0.065	31974946	10p11.22	
rs12243354	10	TET1	A/G	0.125	0.131	70411536	10q21.3	Intron (boundary)
rs7923700	10	GRID1	G/A	0.162	0.190	87843290	10q23.1	Intron
rs2239153	12	VWF	C/T	0.338	0.399	6186667	12p13.31	Intron
rs7303062	12		A/G	0.074	0.084	22990450	12p12.1	
rs10879780	12		T/G	0.235	0.226	74837984	12q21.1	
rs1316571	13		T/C	0.081	0.095	68320718	13q21.32	
rs9550256	13	FAM70B	A/T	0.265	0.220	114494675	13q34	Intron
rs17435983	14		A/G	0.169	0.101	27860597	14q12	
rs8007744	14		G/A	0.265	0.262	28329396	14q12	
rs17777329	14		G/A	0.081	0.060	101934762	14q32.31	
rs4787426	16	IL4R	G/T	0.059	0.065	27384731	16p12.1	Downstream
rs1075355	16	VAT1L	C/G	0.147	0.107	77874149	16q23.1	Intron
rs12931468	16	ATP2C2	G/C	0.074	0.054	84495301	16q24.1	Intron (boundary)
rs8067836	17	LASP1	G/T	0.081	0.071	37081707	17q12	Downstream
rs16955841	17	HLF	G/A	0.105	0.107	53364146	17q22	Intron
rs12450240	17	NARF	T/G	0.265	0.235	80423712	17q25.3	Intron
rs9961715	18	DLGAP1	C/T	0.029	0.054	3824312	18p11.31	Intron
rs12606093	18	KIAA0427	C/A	0.044	0.065	46077295	18q21.1	Intron
rs6074799	20	MACROD2	G/C	0.110	0.101	14771472	20p12.1	Intron
rs9617661	22	TUBA8	G/T	0.029	0.060	18595352	22q11.21	Intron
rs5758913	22		C/T	0.154	0.161	43148259	22q13.2	

Notes: A/B stands for minor/major alleles on the entire sample frequencies.

Of the 77 SNPs, 68 were qualified after Sequenom MassARRAY Assay Design. In a second experiment, we confirmed the results of the first experiment by genotyping the 68 SNPs in 199 HAPE patients and 304 controls of Han Chinese descent. Table 3 summarizes the characteristics of the tested SNPs and their predicted associations with HAPE risk in crude analysis. Three SNPs (rs17484974, rs725050, and rs10178082) were excluded at the 5% *p-value* for Hardy-Weinberg equilibrium (HWE). A χ^2^ test revealed that two SNPs, rs10789097 (OR = 1.825; 95% CI= 1.062–3.135, *p* = 0.027), and rs17777329 (OR = 1.800; 95% CI = 1.083–2.991, *p* = 0.022) were associated with an increased risk of HAPE (Table 3).

**Table 3:** Allele frequencies in cases and controls and odds ratio estimates for HAPE for the replication stage

SNP ID	Gene (s)	Alleles	MAF		HWE	ORs	95% CI		*p*-value
		A^a^/B	Case	Control	*p*-value				
rs4908427	CAMTA1	G/A	0.035	0.048	1	0.728	0.380	1.395	0.337
rs9661274		G/A	0.065	0.067	0.3787	0.967	0.581	1.607	0.896
rs17484974		C/T	0.111	0.112	7.851E-47^#^	0.987	0.660	1.476	0.949
rs12406517	PPAP2B	G/C	0.055	0.054	0.2131	1.020	0.585	1.776	0.946
rs1694212		T/C	0.139	0.133	0.8025	1.049	0.726	1.517	0.798
rs10789097		C/G	0.075	0.043	1	1.825	1.062	3.135	0.027*
rs17188846	KCNH1	C/G	0.139	0.130	0.198	1.080	0.746	1.564	0.683
rs2577156	EPRS	C/A	0.076	0.077	0.08963	0.978	0.607	1.576	0.928
rs3008613	MIA3	G/A	0.093	0.105	0.552	0.871	0.569	1.334	0.526
rs4491711	RASGRP3	G/A	0.063	0.097	1	0.624	0.384	1.014	0.055
rs11125567	CCDC88A	A/G	0.111	0.127	1	0.857	0.578	1.271	0.443
rs11898268		C/A	0.003	0.000	1	-	-	-	-
rs10167840		T/G	0.088	0.095	1	0.914	0.589	1.420	0.690
rs7612512		G/C	0.151	0.172	1	0.857	0.606	1.212	0.382
rs1846594		C/T	0.193	0.232	0.6287	0.794	0.582	1.085	0.148
rs11924340		A/G	0.111	0.086	0.4683	1.329	0.871	2.029	0.186
rs12504325	C4orf6	A/G	0.083	0.090	0.4886	0.909	0.579	1.428	0.679
rs17598758		G/T	0.101	0.105	1	0.950	0.626	1.441	0.808
rs7677143		C/T	0.143	0.191	1	0.709	0.502	1.002	0.051
rs6535838		A/C	0.133	0.137	0.4691	0.972	0.671	1.407	0.879
rs7688505		T/A	0.156	0.140	0.8133	1.135	0.796	1.619	0.483
rs41417552	CMBL	G/A	0.169	0.127	0.4419	1.404	0.985	2.003	0.060
rs2161592		A/G	0.108	0.102	0.7525	1.067	0.707	1.609	0.758
rs3777207	ELL2	A/G	0.108	0.107	0.5511	1.012	0.673	1.521	0.955
rs6595114		C/T	0.118	0.097	0.5042	1.246	0.830	1.870	0.288
rs2193963		C/T	0.106	0.095	1	1.130	0.742	1.721	0.568
rs17652561	SLC6A7	A/G	0.145	0.151	0.6541	0.949	0.663	1.357	0.773
rs2937582		A/G	0.439	0.434	0.6413	1.021	0.791	1.318	0.871
rs2984100		C/G	0.184	0.156	0.2747	1.220	0.873	1.707	0.244
rs7762263		T/C	0.111	0.123	0.594	0.883	0.595	1.312	0.539
rs4715938		G/C	0.161	0.155	0.3873	1.048	0.741	1.481	0.792
rs725050		C/T	0.249	0.243	0.04164^#^	1.029	0.767	1.381	0.849
rs1419722	EIF3B	C/T	0.143	0.149	0.648	0.958	0.669	1.372	0.816
rs10178082		T/A	0.141	0.161	0.0001595^#^	0.852	0.597	1.216	0.378
rs4947936		C/A	0.163	0.150	0.6502	1.109	0.784	1.569	0.559
rs12226072		A/T	0.317	0.340	0.7994	0.897	0.685	1.175	0.431
rs2956956	DLGAP2	C/T	0.078	0.092	0.7288	0.833	0.527	1.317	0.433
rs2980508	SGK223	C/T	0.146	0.135	1	1.097	0.763	1.576	0.619
rs310282		C/A	0.096	0.135	0.3231	0.678	0.451	1.019	0.061
rs4573320		C/T	0.279	0.299	0.8912	0.907	0.685	1.200	0.493
rs1568828	PREX2	A/G	0.108	0.109	0.3729	0.995	0.662	1.494	0.980
rs1006698	KCNV2	T/G	0.216	0.263	0.2387	0.772	0.572	1.041	0.089
rs1011531		A/G	0.118	0.120	1	0.981	0.664	1.450	0.925
rs13289064		C/G	0.231	0.183	1	1.346	0.986	1.837	0.060
rs10984811	ANP32B	C/A	0.178	0.148	0.6479	1.250	0.889	1.757	0.199
rs12554842	COL5A1	T/C	0.095	0.109	0.2273	0.867	0.569	1.320	0.505
rs11593009		T/A	0.078	0.076	0.6833	1.032	0.642	1.658	0.896
rs12243354	TET1	A/G	0.138	0.137	0.809	1.014	0.703	1.464	0.940
rs7923700	GRID1	G/A	0.116	0.117	0.399	0.988	0.666	1.467	0.954
rs2239153	VWF	C/T	0.415	0.428	0.4145	0.948	0.734	1.224	0.682
rs7303062		A/G	0.050	0.061	0.6129	0.817	0.467	1.428	0.477
rs10879780		T/G	0.193	0.192	0.5762	1.007	0.731	1.389	0.964
rs1316571		T/C	0.078	0.079	0.102	0.985	0.616	1.577	0.951
rs9550256	FAM70B	A/T	0.234	0.261	0.232	0.865	0.644	1.161	0.333
rs17435983		A/G	0.143	0.128	0.7988	1.136	0.786	1.640	0.497
rs8007744		G/A	0.261	0.267	0.7692	0.973	0.729	1.299	0.852
rs17777329		G/A	0.085	0.049	0.5287	1.800	1.083	2.991	0.022*
rs4787426	IL4R	G/T	0.083	0.066	1	1.284	0.795	2.073	0.306
rs1075355	VAT1L	C/G	0.131	0.092	0.1553	1.481	0.992	2.212	0.053
rs12931468	ATP2C2	G/C	0.055	0.044	1	1.259	0.707	2.244	0.434
rs8067836	LASP1	G/T	0.111	0.082	0.7057	1.387	0.906	2.125	0.131
rs16955841	HLF	G/A	0.133	0.109	0.1143	1.255	0.830	1.898	0.282
rs12450240	NARF	T/G	0.242	0.281	0.8872	0.818	0.612	1.093	0.174
rs9961715	DLGAP1	C/T	0.055	0.041	0.4021	1.364	0.758	2.455	0.298
rs12606093	KIAA0427	C/A	0.063	0.066	0.3739	0.952	0.568	1.595	0.851
rs6074799	MACROD2	G/C	0.113	0.140	0.2313	0.784	0.533	1.154	0.216
rs9617661	TUBA8	G/T	0.050	0.033	1	1.556	0.826	2.930	0.168
rs5758913		C/T	0.156	0.151	0.6541	1.035	0.729	1.469	0.848

Notes: a Minor allele; *p value ≤ 0.05 indicates statistical significance; ^#^site with HWE *p* ≤ 0.05 is excluded;

Abbreviations: HWE, Hardy-Weinberg Equilibrium; MAF, minor allele frequency; SNP, single nucleotide polymorphism; ORs, odds ratios; CI, confidence interval.

Associations between the SNPs and HAPE risk were tested under five different genetic models (co-dominant, dominant, co-dominant, recessive, and log-additive). Seven SNPs were associated with HAPE susceptibility. The rs41417552 SNP was associated with an increased risk of HAPE based on the results of the co-dominant (OR = 1.58; 95% CI = 1.04–2.40, *p* = 0.057 for the “A/G” genotype), dominant (OR = 1.62; 95% CI = 1.07–2.44, *p* = 0.022 for the “A/G-G/G” genotype), over-dominant (OR = 1.87; 95% CI = 1.06–3.27, *p* = 0.03 for the “A/G” genotype), and log-additive (OR = 1.59; 95% CI = 1.09–2.32, *p* = 0.017) models. The rs10984811 SNP increased HAPE risk in both the co-dominant (OR = 3.95; 95% CI = 1.33–11.73, *p* = 0.032 for the “C/C” genotype) and recessive (OR= 3.97; 95% CI = 1.34–11.75, *p* = 0.0089 for the “C/C” genotype) models. The rs17777329 SNP was also associated with an increased risk of HAPE in the co-dominant (OR = 1.88; 95% CI = 1.07–3.30, *p* = 0.051), dominant (OR = 1.95; 95% CI = 1.12–3.37, *p* = 0.018), over-dominant (OR = 1.87; 95% CI = 1.06–3.27, *p* = 0.03), and log-additive (OR = 1.89; 95% CI = 1.13–3.16, *p* = 0.015) models. The rs1075355 SNP was associated with increased HAPE risk in the co-dominant (OR = 1.65; 95% CI = 1.04–2.62, *p* = 0.093) and over-dominant (OR = 1.66; 95% CI = 1.04–2.63, *p* = 0.032) models. Additionally, the rs12226072 (OR = 0.58; 95% CI = 0.40–0.86, *p* = 0.0053) and rs6074799 (OR = 0.59; 95% CI = 0.37–0.93, *p* = 0.02) SNPs were associated with a decreased risk of HAPE in the over-dominant model, and the rs7677143 SNP was associated with a decreased risk of HAPE in the log-additive model (OR = 0.69; 95% CI = 0.48–0.99, *p* = 0.039) (Table 4).

**Table 4:** Logistic regression analysis of the associations between SNPs and HAPE risk

SNP	Model	Genotype	Controls	Cases	OR (95 % CI)^a^	*P*-value^a^	AIC	BIC
rs7677143	Co-dominant	T/T	199 (65.5%)	145 (72.9%)	1	0.11	661.6	682.7
		T/C	94 (30.9%)	51 (25.6%)	0.72 (0.48–1.09)			
		C/C	11 (3.6%)	3 (1.5%)	0.38 (0.10–1.40)			
	Dominant	T/T	199 (65.5%)	145 (72.9%)	1	0.062	660.6	677.5
		T/C-C/C	105 (34.5%)	54 (27.1%)	0.69 (0.46–1.02)			
	Recessive	T/T-T/C	293 (96.4%)	196 (98.5%)	1	0.16	662.1	678.9
		C/C	11 (3.6%)	3 (1.5%)	0.41 (0.11–1.53)			
	Over-dominant	T/T-C/C	210 (69.1%)	148 (74.4%)	1	0.16	662.1	678.9
		T/C	94 (30.9%)	51 (25.6%)	0.75 (0.50–1.12)			
	Log-additive	—	—	—	**0.69 (0.48–0.99)**	0.039	659.8	676.7
rs12226072	Co-dominant	T/T	131 (43.1%)	103 (51.8%)	1	0.017	657.9	679
		A/T	139 (45.7%)	66 (33.2%)	**0.61 (0.41–0.90)**			
		A/A	34 (11.2%)	30 (15.1%)	1.19 (0.67–2.09)			
	Dominant	T/T	131 (43.1%)	103 (51.8%)	1	0.077	660.9	677.8
		A/T-A/A	173 (56.9%)	96 (48.2%)	0.72 (0.50–1.04)			
	Recessive	T/T-A/T	270 (88.8%)	169 (84.9%)	1	0.16	662	678.9
		A/A	34 (11.2%)	30 (15.1%)	1.48 (0.86–2.54)			
	Over-dominant	T/T-A/A	165 (54.3%)	133 (66.8%)	1	0.0053	656.3	673.2
		A/T	139 (45.7%)	66 (33.2%)	**0.58 (0.40–0.86)**			
	Log-additive	—	—	—	0.92 (0.71–1.20)	0.56	663.7	680.6
rs6074799	Co-dominant	C/C	222 (73%)	159 (79.9%)	1	0.03	659	680.1
		C/G	79 (26%)	35 (17.6%)	**0.60 (0.38–0.95)**			
		G/G	3 (1%)	5 (2.5%)	2.57 (0.59–11.13)			
	Dominant	C/C	222 (73%)	159 (79.9%)	1	0.068	660.7	677.6
		C/G-G/G	82 (27%)	40 (20.1%)	0.67 (0.43–1.04)			
	Recessive	C/C-C/G	301 (99%)	194 (97.5%)	1	0.15	662	678.9
		G/G	3 (1%)	5 (2.5%)	2.88 (0.67–12.40)			
	Over-dominant	C/C-G/G	225 (74%)	164 (82.4%)	1	0.02	658.7	675.6
		C/G	79 (26%)	35 (17.6%)	**0.59 (0.37–0.93)**			
	Log-additive	—	—	—	0.78 (0.52–1.15)	0.21	662.5	679.4
rs41417552	Co-dominant	A/A	230 (75.7%)	135 (68.2%)	1	0.057	658.3	679.4
		A/G	71 (23.4%)	59 (29.8%)	**1.58 (1.04–2.40)**			
		G/G	3 (1%)	4 (2%)	2.68 (0.58–12.38)			
	Dominant	A/A	230 (75.7%)	135 (68.2%)	1	0.022	656.7	673.6
		A/G-G/G	74 (24.3%)	63 (31.8%)	**1.62 (1.07–2.44)**			
	Recessive	A/A-A/G	301 (99%)	194 (98%)	1	0.27	660.8	677.7
		G/G	3 (1%)	4 (2%)	2.35 (0.51–10.80)			
	Over-dominant	A/A-G/G	233 (76.6%)	139 (70.2%)	1	0.042	657.9	674.8
		A/G	71 (23.4%)	59 (29.8%)	**1.54 (1.02–2.34)**			
	Log-additive	—	—	—	**1.59 (1.09–2.32)**	0.017	656.3	673.2
rs10984811	Co-dominant	A/A	219 (72%)	139 (69.8%)	1	0.032	659.2	680.3
		C/A	80 (26.3%)	49 (24.6%)	0.97 (0.64–1.49)			
		C/C	5 (1.6%)	11 (5.5%)	**3.95 (1.33–11.73)**			
	Dominant	A/A	219 (72%)	139 (69.8%)	1	0.52	663.7	680.5
		C/A-C/C	85 (28%)	60 (30.1%)	1.14 (0.76–1.70)			
	Recessive	A/A-C/A	299 (98.4%)	188 (94.5%)	1	0.0089	657.2	674.1
		C/C	5 (1.6%)	11 (5.5%)	**3.97 (1.34–11.75)**			
	Over-dominant	A/A-C/C	224 (73.7%)	150 (75.4%)	1	0.68	663.9	680.8
		C/A	80 (26.3%)	49 (24.6%)	0.92 (0.60–1.39)			
	Log-additive	—	—	—	1.28 (0.91–1.80)	0.16	662	678.9
rs17777329	Co-dominant	A/A	275 (90.5%)	167 (83.9%)	1	0.051	660.1	681.2
		A/G	28 (9.2%)	30 (15.1%)	**1.88 (1.07–3.30)**			
		G/G	1 (0.3%)	2 (1%)	3.69 (0.33–41.57)			
	Dominant	A/A	275 (90.5%)	167 (83.9%)	1	0.018	658.4	675.3
		A/G-G/G	29 (9.5%)	32 (16.1%)	**1.95 (1.12–3.37)**			
	Recessive	A/A-A/G	303 (99.7%)	197 (99%)	1	0.3	663	679.9
		G/G	1 (0.3%)	2 (1%)	3.40 (0.30–38.26)			
	Over-dominant	A/A-G/G	276 (90.8%)	169 (84.9%)	1	0.03	659.3	676.2
		A/G	28 (9.2%)	30 (15.1%)	**1.87 (1.06–3.27)**			
	Log-additive	—	—	—	**1.89 (1.13–3.16)**	0.015	658.1	675
rs1075355	Co-dominant	G/G	253 (83.2%)	149 (74.9%)	1	0.093	661.3	682.4
		G/C	46 (15.1%)	48 (24.1%)	**1.65 (1.04–2.62)**			
		C/C	5 (1.6%)	2 (1%)	0.71 (0.13–3.85)			
	Dominant	G/G	253 (83.2%)	149 (74.9%)	1	0.052	660.3	677.2
		G/C-C/C	51 (16.8%)	50 (25.1%)	1.56 (1.00–2.45)			
	Recessive	G/G-G/C	299 (98.4%)	197 (99%)	1	0.6	663.8	680.7
		C/C	5 (1.6%)	2 (1%)	0.64 (0.12–3.51)			
	Over-dominant	G/G-C/C	258 (84.9%)	151 (75.9%)	1	0.032	659.5	676.4
		G/C	46 (15.1%)	48 (24.1%)	**1.66 (1.04–2.63)**			
	Log-additive	—	—	—	1.40 (0.93–2.11)	0.1	661.4	678.3

Notes: ^a^Adjusted for age and sex. *P-value ≤ 0.05 indicates statistical significance.

Abbreviations: SNP, single nucleotide polymorphism; OR, odds ratio; CI, confidence interval; AIC, Akaike's Information Criterion; BIC, Bayesian Information Criterion.

To identify genes that might be involved in HAPE invasion, we also performed gene annotation and functional classification for the 7 significant loci we identified in the replication study. GO and KEGG pathway enrichment analyses identified 5 potential candidate genes located within ± 500kb of these SNPs (Table 5). These genes were mainly involved in cellular tight junctions, oxidation and reduction, extracellular matrix metabolism, pulmonary development, and pulmonary smooth muscular tension adjustment.

**Table 5:** Go and pathway analysis of the top genes of GWAS

**Function**	***p*-value**	**Adjusted *p*-value**	**Genes**
zinc ion binding	7.99E-07	1.60E-06	*ADAMTS18;VAT1L*
protein binding	1.59E-05	1.06E-05	*INADL;KCNV2*
thyroxine 5-deiodinase activity	7.56E-05	3.36E-05	*DIO3*
very-low-density lipoprotein receptor activity	2.27E-04	6.52E-05	*VLDLR*
thyroxine 5′-deiodinase activity	2.27E-04	6.52E-05	*DIO3*
metal ion binding	2.45E-04	6.52E-05	*ADAMTS18*
low density lipoprotein receptor activity	8.31E-04	1.45E-04	*VLDLR*
peptidase activity	8.69E-04	1.45E-04	*ADAMTS18*
oxidoreductase activity	0.001153	1.58E-04	*WWOX*
selenium binding	0.002265	2.01E-04	*DIO3*
ATP binding	0.004538	3.70E-04	*CCT5*
voltage-gated potassium channel activity	0.007231	5.26E-04	*KCNV2*
metalloendopeptidase activity	0.007756	5.35E-04	*ADAMTS18*
unfolded protein binding	0.008356	5.52E-04	*CCT5*
potassium ion binding	0.00948	6.12E-04	*KCNV2*
nucleotide binding	0.010312	6.34E-04	*CCT5*
manganese ion binding	0.011276	6.63E-04	*NUDT7*
coenzyme binding	0.0115	6.67E-04	*WWOX*
hydrolase activity	0.012359	6.83E-04	*NUDT7*
voltage-gated ion channel activity	0.013964	7.35E-04	*KCNV2*
protein dimerization activity	0.031265	0.001374	*WWOX*
magnesium ion binding	0.032144	0.001398	*NUDT7*
hydrolase activity, acting on acid anhydrides, in phosphorus-containing anhydrides	0.053236	0.002117	*NUDT7*
calcium ion binding	0.06698	0.002528	*VLDLR*
receptor activity	0.121001	0.00436	*VLDLR*
**Pathways**	***p*-value**	**Adjusted *p*-value**	**Genes**
1,4-Dichlorobenzene degradation	0.000371	0.000742	CMBL
gamma-Hexachlorocyclohexane degradation	0.006661	0.001665	CMBL
Maturity onset diabetes of the young	0.008871	0.001971	IAPP
Tight junction	0.049301	0.002641	INADL
Wnt signaling pathway	0.054948	0.002641	PPP2R5C

## DISCUSSION

In this study, we conducted a two-stage GWAS analysis to investigate the genetic factors associated with HAPE in a Han Chinese population. Seven loci, including four susceptibility loci and three protective loci, were found to be associated with HAPE in this analysis. Gene annotation and functional classification of these loci revealed that five of the candidate genes are potentially involved in the pathogenesis of HAPE. To the best of our knowledge, this is one of the largest studies to explore the genetic factors underlying the development of HAPE in a Han Chinese population.

The rs10789097 locus contained no annotated genes, and the gene nearest to it was INADL, which encodes inactivation no-after potential (INAD) protein, also known as protein associated with Lin seven 1 (Pals1) -associated tight junction protein (PATJ). INAD contains multiple PDZ domains, which are protein-protein interaction modules that typically bind to short peptide sequences at the carboxyl terminus of target proteins. Proteins containing multiple PDZ domains often bind to different transmembrane and intracellular proteins and play central roles as organizers of multimeric complexes [15]. PATJ is a polarity protein and plays a complex role in the maintenance of epithelial polarity [16]. Considering that stress failure in pulmonary capillaries is an important contributor to HAPE pathogenesis, we speculate that the INADL gene may also impact the pathogenesis of HAPE.

The KCNV2 gene, which encodes the Kcnv2 protein, belongs to a group of potassium channel modulatory subunits that are electrically silent and cannot form functional homotetramers. These silent subunits form heterotetramers that modulate the properties of other subunits, increasing the functional diversity of channel subfamilies [17]. Voltage-gated K^+^ (K_V_) channel activity in pulmonary artery smooth muscle cells (PASMC) is important for the control of apoptosis and proliferation as well as the regulation of membrane potential and pulmonary vascular tone [18]. A previous study demonstrated that KNCV2 contributes to susceptibility to and was considered a genetic modifier of epilepsy [17]. However, the role of KNCV2 in HAPE remains unknown, and additional studies are needed.

The rs1075355 SNP had the strongest association in this study. It is located in the intron of the VAT1L gene and encodes a vesicle amine transport 1 homologue; its cellular localization and functions have not yet been researched. An association study suggested that a locus on chromosome 16q23-24 (including VAT1L) affected HDLC levels in two independent French-Canadian populations [19]. Additionally, a genome-wide association study of the rate of cognitive decline in Alzheimer's disease indicated that rs9934540 genetic variants in the VAT1L gene intron were positively associated with the development of Alzheimer's disease [20]. Two different genes, ADAMTS18 and WWOX, are adjacent to the rs1075355 SNP.

ADAMTS18 is a member of the ADAMTS protease family, which is comprised of complex secreted enzymes containing a reprolysin-type prometalloprotease domain attached to an ancillary domain with a highly-conserved structure including at least one thrombospondin type 1 repeat. Known functions of ADAMTS proteases include processing procollagens and von Willebrand factor and catabolism of aggrecan, versican, and brevican. ADAMTS also play important roles in connective tissue organization, coagulation, inflammation, arthritis, angiogenesis, and cell migration [21, 22] and are regulated by the Tissue Inhibitor of Metalloproteinase 3 Gene (TIMP3). Furthermore, Kobayashi *et al*.'s study in a Japanese population demonstrated that TIMP3 was associated with susceptibility to HAPE [23]. TIMPs play a crucial role in the physiological turnover of the extracellular matrix (ECM) by tightly regulating matrix metalloproteinase (MMP) activity [24]. TIMP3 is the only TIMP that binds tightly to the ECM, and the balance between MMPs and TIMPs plays an important role in maintaining the integrity of healthy tissues. Disturbances of the TIMP/MMP system are implicated in various pathologic conditions in lungs, including pulmonary inflammation, edema, emphysema, and fibrosis, where loss of ECM integrity is a principal feature [25]. Our findings together with those of previous studies demonstrate that the balance between MMPs and TIMPs plays an important role in HAPE pathogenesis.

The human WWOX gene encodes a putative tumor suppressor WW domain-containing oxidoreductase WOX1 (also known as WWOX or FOR). High frequencies of loss of heterozygosity (LOH) in this gene have been observed in prostate, lung, breast, and other cancers [27]. A recent genome-wide association analysis identified WWOX as one of the loci associated with forced vital capacity (FVC), a spirometric measure of pulmonary function used to diagnose and monitor lung diseases [27]. These findings indicate that the WWOX gene may be involved in lung development and the pathogenesis of restrictive lung disease; future studies are needed to determine whether WWOX is similarly associated with HAPE pathogenesis.

Although the statistical power of the present study was sufficient, some limitations should be considered when interpreting these results. First, the patient sample sizes were relatively small, and the association between the identified polymorphisms and HAPE susceptibility should be confirmed in future studies with larger sample sizes. Secondly, the mechanisms by which the potential candidate genes contribute to the pathogenesis of HAPE remain unclear, and functional studies of these candidate genes are needed. In conclusion, our study provides new evidence regarding the pathogenesis of HAPE in the Han Chinese population. Although the genetic factors that contribute to the development of HAPE remain largely unknown, we identified candidate genes that contribute to HAPE susceptibility. However, polymorphisms in these genes should be examined further before definitive conclusions regarding their role in HAPE pathogenesis can be made.

## MATERIALS AND METHODS

### Study populations

In this two-stage case-control study, we evaluated associations between genetic variants across the human genome and the risk of HAPE. All participants included in the study were from the Han Chinese population. Study subjects for both GWAS scan of HAPE and the replication phase of the experiment were selected according to detailed inclusion and exclusion criteria. Briefly, patients who lived on the Tibet Plateau and were diagnosed with HAPE were recruited from the General Hospital of Tibet Military Region. Control subjects were Han Chinese immigrants living in Lhasa, Tibet, and their medical histories and physical examinations confirmed that they were in good health. Demographic information was collected through interviews using a standard questionnaire. Ultimately, 267 HAPE cases (89 recurrent HAPE cases; mean age 32.6 ± 10.7 years) and 304 controls (mean age 36.2 ± 4.5 years) were selected for the study. Two mL of venous blood were collected from each individual into tubes containing 2% EDTA-K2, centrifuged, and stored at –80°C until analysis. DNA was extracted from whole blood samples using the QIAamp^®^ DNA Blood Mini kit (Qiagen), and DNA concentrations were measured using a NanoDrop 2000. Informed consent was obtained from all subjects, and the Human Ethics Committee of our institute approved the investigation.

### Study design

For the GWAS scan experiment, we scanned DNA samples from 68 patients with recurrent HAPE using Affymetrix SNP Array 6.0 Chips. The allele frequencies of the 68 patients were then compared to those of 84 HapMap CHB subjects to identify significant differences in SNP frequencies. In the replication experiment, associations between the SNPs identified in the GWAS scan and risk of HAPE where examined in 199 HAPE patients and 304 unrelated healthy controls. Furthermore, to identify candidate genes that might underlie HAPE susceptibility, we conducted Gene Ontology (GO) and Kyoto Encyclopedia of Genes and Genomes (KEGG) pathway enrichment analysis for the genes involved in the associated genetic loci.

### Quality control (QC) in GWAS

A total of 906,660 SNPs were genotyped in 68 patients with recurrent HAPE during the GWAS experiment using Affymetrix Genome-Wide Human SNP Array 6.0 Chips as described previously [28]. A systematic quality control (QC) procedure was applied to both SNPs and samples prior to the association analysis. SNPs were excluded if they (i) did not map onto autosomal chromosomes; (ii) had a call rate of less than 95%; (iii) had a minor allele frequency (MAF) less than 0.05; or (iv) deviated from Hardy-Weinberg equilibrium (*p* < 0.001). Sixty-eight HAPE cases and 84 controls with 502,689 SNPs remained after QC.

### SNP selection and genotyping in the replication study

After genome-wide association analysis, we compared the allele frequencies of the 502,689 SNPs in the 68 recurrent HAPE cases to those in the 84 HapMap CHB controls using a chi-squared (χ^2^) test. Allele frequencies differed significantly between HAPE cases and controls for 77 SNPs. In the replication study, these 77 SNPs were genotyped in 199 HAPE patients and 304 normal controls. SNPs that were significantly associated with HAPE risk (*p* < 0.05) in the replication study were selected for GO and KEGG pathway enrichment analyses. Genotyping was performed using Sequenom MassARRAY Assay Design 3.0 Software [29] with a genotype success rate greater than 97.3%.

### Statistical analysis

SPSS 17.0 statistical software was used for statistical analysis. An exact test was used to test the departure of each SNP frequency from Hardy–Weinberg equilibrium (HWE) in control subjects. Differences in SNP genotype distribution between HAPE patients and controls were compared using a χ^2^ test [30]. Odds ratios (ORs) and 95% confidence intervals (CIs) were determined using unconditional logistic regression analysis with adjustments for age and gender [31]. All *p* values presented in this study are two-sided; *p* < 0.05 indicated a statistically significant difference.

Associations between SNPs and HAPE risk were tested using various genetic models (co-dominant, dominant, over-dominant, recessive, and log-additive) and analyzed using SNP Stats software (obtained from http://bioinfo.iconcologia.net, Catalan Institute of Oncology, Barcelona, Spain). To reduce population stratification, a multidimensional scaling approach (MDS) was used and a QQ-plot was generated using PLINK software (version 1.07) (http://www.cog-genomics.org/plink2/) [32]. R software (version 2.11.1) was used for statistical analysis and to generate plots, including Manhattan plots. GO analysis were performed using Bingo software [33], and pathway enrichment analyses were performed using Mas 3.0 software (http://bioinfo.capitalbio.com/mas3/).
